# A Cobb Douglas Stochastic Frontier Model on Measuring Domestic Bank Efficiency in Malaysia

**DOI:** 10.1371/journal.pone.0042215

**Published:** 2012-08-10

**Authors:** Md. Zobaer Hasan, Anton Abdulbasah Kamil, Adli Mustafa, Md. Azizul Baten

**Affiliations:** 1 Mathematics Section, School of Distance Education, Universiti Sains Malaysia, Penang, Malaysia; 2 School of Mathematical Sciences, Universiti Sains Malaysia, Penang, Malaysia; 3 Department of Decision Science, School of Quantitative Sciences, Universiti Utara Malaysia, Darul Aman, Malaysia; Universidad Veracruzana, Mexico

## Abstract

Banking system plays an important role in the economic development of any country. Domestic banks, which are the main components of the banking system, have to be efficient; otherwise, they may create obstacle in the process of development in any economy. This study examines the technical efficiency of the Malaysian domestic banks listed in the Kuala Lumpur Stock Exchange (KLSE) market over the period 2005–2010. A parametric approach, Stochastic Frontier Approach (SFA), is used in this analysis. The findings show that Malaysian domestic banks have exhibited an average overall efficiency of 94 percent, implying that sample banks have wasted an average of 6 percent of their inputs. Among the banks, RHBCAP is found to be highly efficient with a score of 0.986 and PBBANK is noted to have the lowest efficiency with a score of 0.918. The results also show that the level of efficiency has increased during the period of study, and that the technical efficiency effect has fluctuated considerably over time.

## Introduction

Bank's performance measurement and assessment are one of the most important agendas in today's business world. Failure to do some satisfactory performance may damage the bank's reputation, leading to customer defections and breakdowns with other key stakeholders, such as deterioration or loss of investor confidence in management. Thus, banks not only need to be profitable, but also efficient; otherwise, it may create instability and obstacle in the process of development in any economy.

The objective of this paper is to investigate the level of technical efficiency of the domestic banks in Malaysia, which are listed in the Kuala Lumpur Stock Exchange (KLSE). Selection of Stochastic Frontier Analysis (SFA) approach or Data Envelopment Analysis (DEA) approach for measuring efficiency is controversial [1]. In the present study, the parametric SFA approach has been employed to estimate the technical efficiency of Malaysian domestic banks for the period 2005–2010. The reason for using the SFA approach, instead of the DEA approach, in this study is as follows: Addition of a variable in the DEA model leads to an extra constraint, which affects the DEA efficiency results, even though the added variable may be statistically insignificant in the SFA model.

SFA employs a composed error model in which inefficiencies are assumed to follow an asymmetric distribution, usually the half-normal, while random errors are assumed to follow a symmetric distribution, usually the standard normal [2]. Most past studies used the half-normal and truncated normal distribution as assumption on the inefficiency effects model because of the ease of estimation and interpretation [3].

### Literature Review

In the banking literature, two major methods for the empirical estimation of bank efficiency are often used: parametric and nonparametric approaches; however, there is no accord regarding which of the major approach is superior [4]. The methods used in parametric approach are SFA, Thick Frontier Approach (TFA), and DFA. On the other hand, the nonparametric researches use Data Envelopment Analysis (DEA), Malmquist Index, Tornqvist Index, and Distance Functions to measure bank efficiency. In the parametric studies, SFA is often used. In the nonparametric, DEA is the extensively used method.

Studies on efficiency of banking using stochastic frontier approaches did not start until the authors of [5] started their own. They applied the frontier approach to banking industry by focusing on the operating efficiency of the branches of a savings bank. Since then, many studies had been carried out using frontier approaches to measure banking efficiency. Past studies on bank efficiency and other financial institutions had focused mainly on the USA [6]–[8] and other developed countries [9], such as Australia [10], Spain [11], Norway [12], and Italy [13]. While the large majority of bank efficiency studies have been based on the banking data of developed countries, in recent years, researchers have started to examine the efficiency of banks in developing countries [14]–[22]. A few studies examining the bank efficiency in Malaysia have been carried out [23]–[24].

## Materials and Methods

### Theoretical Stochastic Frontier Model

Technical efficiency (TE) has two types of measures: output-oriented and input-oriented. If it is an output-oriented measure, then TE is a bank's ability to make maximum output, given its sets of inputs. If it is an input-oriented measure, then TE measure reflects the degree to which a bank could reduce its inputs used in the production of given outputs. We have adopted an output-oriented measure in our study.

There are various methods of measuring technical efficiency [25]–[27]. In the present study, we have used the approach proposed by [28], which explicitly accounts for statistical noise. The specification of the model may be expressed as:
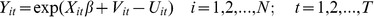
(1)where 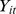 denotes the output for the *i*th bank in the *t*th time period; 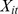 is a vector whose values are functions of inputs for the *i*th bank in the *t*th time period; 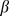 is a vector of unknown parameters to be estimated; 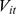 are assumed to be independent and identically distributed random errors which have normal distribution with mean zero and unknown variance 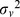 and also independent of 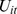; and 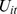 are non-negative unobservable random variables associated with the technical inefficiency of production. The assumption that the 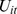 and the 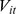 are independently distributed for all *t* = 1,2,…,*T* and *i* = 1,2,…,*N*, is obviously a simplifying, but restrictive, condition.

Now, the technical inefficiency effect 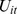 is defined by [28] as:
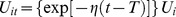
where *η* is an unknown scalar parameter to be estimated, which determines whether inefficiencies are time-varying or time-invariant. If *η* is positive, then the technical inefficiencies of banks decline over time. If *η* is zero, then the technical inefficiencies of banks remain constant. However, if *η* is negative, then the technical inefficiencies of companies increase over time. *U_i_, i* = 1,2,…,*N* are independent and identically distributed with unknown mean *μ* and unknown variance 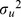.

Thus, the technical efficiency for the *i*th bank in the *t*th year can be defined in the context of stochastic frontier model (1) as follows [28]:
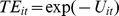
where *U_it_* denotes the specifications of the inefficiency model in (2). This measure is done with the calculation of maximum-likelihood estimates for the parameters of the stochastic frontier model by using the computer program FRONTIER Version 4.1 [29].

### Measurement of Variables

One of the crucial debated issues in banking literature is output measurement. Under production approach, the output is measured by number and type of transactions or accounts. As only physical inputs are needed to provide financial services, inputs use only physical units, such as labor and capital. Under the intermediation approach, banks are treated as financial intermediaries that combine deposits, labor, and capital to produce loans and investments. The values of loans and investments are treated as output measures; labor, deposits, and capital are inputs; and operating costs and financial expenses include total cost. The present study has adopted intermediation approach to specify outputs and inputs of the studied banks.

### Data Set

We used data of the period 2005–2010 from six domestic banks in Malaysia listed in the KLSE market. These banks were AMMB, RHBCAP, MAYBANK, PBBANK, AFFIN, and HLBANK. Most of the data were collected from annual reports of the specific banks of Malaysia.

### Dependent Variable

Total Earning Assets: In this study, total earning assets (TEA) were used to represent the dependent variable, which include financing, dealing securities, investment securities, and placements with other banks.

### Independent Variables

Total Deposits: Total deposits (TD) is the input variable that represents deposits from customers and other banks.

Total Overhead Expenses: Total Overhead Expenses (TOE) is the other input variable that represents personnel expenses and other operating expenses.

TIME: To find the productive efficiency of a bank over time, we took time as the input variable. In this study, we collected data of 6 years from 2005 to 2010.

### Empirical Stochastic Frontier Model

The functional form of the Cobb Douglas stochastic frontier production model is defined as:
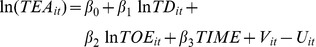
(4)where the subscripts *i* and *t* represent the *i*th bank and *t*th year of observation, and *i* = 1,2,…,6; *t* = 1,2,….,6; *TEA_it_* represents the TEA; *TD_it_* represents the TD; *TOE_it_* represents the TOE; *TIME* represents the year; and “ln” refers to the natural logarithm.

## Results

### Ordinary Least Square Estimates of Cobb Douglas Production Function

Bank efficiency estimates were measured using a Cobb Douglas stochastic frontier production model proposed in [28]. A two-step process was employed to find out the technical efficiency using maximum-likelihood method. The ordinary least square (OLS) estimates of the parameters were obtained by grid search in the first step, and then these estimates were used to estimate the maximum-likelihood estimates of the parameters treated as the frontier estimates of Cobb Douglas stochastic frontier production model. The OLS estimates of the parameters in the model are presented in Table 1. From the analysis, we observed that the coefficient of TD was at 1% level of significance with a value of 1.002, while that of TOE and TIME was insignificant with the values of −0.006 and −0.013, respectively. The parameter σ was positive, which indicates that the observed output differed from frontier output.

**Table 1:** OLS Estimates of Cobb Douglas Production Function.

Variables	Parameters	Coefficients	S.E	*t*-Value
Constant	*β_0_*	0.445@	0.754	0.590
Total deposits	*β_1_*	1.002^*^	0.032	30.868
Total overhead expenses	*β_2_*	-0.006@	0.022	−0.304
Time	*β_3_*	-0.013@	0.011	−1.162
Sigma-squared	*σ^2^*	0.013		

*, **, *** Significance level at 1, 5, and 10%, respectively, @ indicates insignificant, S.E = Standard Error.

### Maximum-Likelihood Estimates of Cobb Douglas Production Function

The maximum-likelihood estimates of the parameters of Cobb Douglas stochastic frontier production model are presented in Table 2. From the analysis, we observed that the coefficients of TD and TIME were at 1 and 5% level of significance, with values of 0.997 and −0.036, respectively, indicating that the TEA (output) was explained by 99% TD and 3% TIME. On the other hand, the coefficient of TOE was found to be insignificant with a value of −0.002, indicating that the output variable was explained only by 0.2% TOE. The coefficient of “total deposits” showed a positive sign, indicating that banks that use more deposits are more productive, whereas the coefficient of “total overhead expenses” showed a negative sign, indicating that banks that use less overhead expenses are more productive. The value of γ was estimated to be 0.034, which demonstrates that 3 percent variations in output among the banks were due to the differences in technical efficiency. It is evident from Table 2 that the estimate of σ is 0.034, which is significantly different from zero, indicating a good fit. As the estimates for the η parameter were observed to be positive, it can be concluded that the technical inefficiency effects tend to decrease over time.

**Table 2:** Maximum-Likelihood Estimates of Cobb Douglas Production Function.

Variables	Parameters	Coefficients	S.E	*t*-Value
Constant	*β_0_*	0.613@	0.393	1.559
Total deposits	*β_1_*	0.997^*^	0.014	67.930
Total overhead expenses	*β_2_*	-0.002@	0.016	−0.146
Time	*β_3_*	−0.036^**^	0.015	−2.271
Sigma-squared	*σ^2^*	0.008^*^	0.003	2.879
Gamma	*γ*	0.034@	0.173	0.196
eta	*η*	0.425^**^	0.168	2.529

*, **, *** Significance level at 1, 5, and 10%, respectively, @ indicates insignificant, S.E = Standard Error.

### Year-wise Mean Efficiency of Banks

A firm is regarded as technically efficient if it can get maximum outputs from given inputs or reduce inputs used in producing given outputs. Therefore, firms on the production frontiers are labeled as “best practice,” and they show optimum efficiency in the utilization of their resources. A value of 1.0 indicates that a firm lies on the best practice frontier or full efficiency. A value of less than 1.0 shows operations below the frontier or inefficient use of resources.

**Figure 1:**
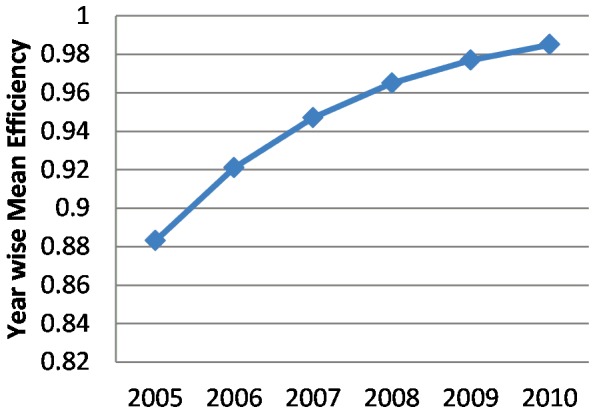
Year-wise mean efficiency.

The year-wise average bank efficiency is illustrated in Table 3 and Figure 1. It could be observed that on an average, banks were 94 percent efficient with respect to the best performing bank during the study period. In other words, the sample banks had wasted an average of 6 percent of their inputs. From this investigation, we also observed that the highest average efficiency was 98.5 percent in 2010, while the lowest average efficiency was 88.3 percent in 2005. Thus, the average technical efficiency score of the studied six banks ranged between 88 and 98 percent, and increased over the years. However, an earlier study [30] found the score ranging between 68 and 80 percent on a decreasing trend, while another study [31] found Malaysian banks exhibiting a score of 95.9 percent. From Figure 1, the overall situation of banks' performance can be clearly understood.

**Table 3:** Year-wise Mean Efficiency of Banks.

Year	Mean
2005	0.883
2006	0.921
2007	0.947
2008	0.965
2009	0.977
2010	0.985
Mean	0.9463

### Year-wise Bank-level Efficiency

**Figure 2:**
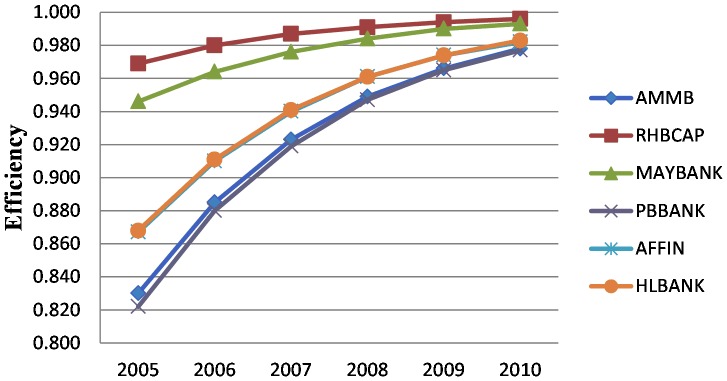
Bank level efficiency over time.

The year-wise bank-level efficiency of six banks is presented in Table 4 and Figure 2. From the efficiency scores presented in Table 4, it can be noted that all the banks' average efficiency is on an increasing trend. The most efficient bank during the study period was RHBCAP (98.6 percent) and the least efficient bank during the data period was PBBANK (91.8 percent). At the beginning of the study period, RHBCAP was the most efficient and retained its place at the end of the period as well. Similarly, PBBANK bank was the least efficient and retained its place at the end of the study period. However, the disparity between the highest efficiency (98.6 percent) and lowest efficiency (91.8 percent) was not very large. During the period of 2005–2010, the efficiency of all six banks was almost stable and consistent over time. AFFIN bank and HLBANK showed almost the same efficiency during the study period. Figure 2 shows a more clear perception about the performance of an individual bank.

**Table 4:** Year-wise Bank level Efficiency.

Year	AMMB	RHBCAP	MAYBANK	PBBANK	AFFIN	HLBANK
2005	0.830	0.969	0.946	0.822	0.867	0.868
2006	0.885	0.980	0.964	0.880	0.910	0.911
2007	0.923	0.987	0.976	0.919	0.940	0.941
2008	0.949	0.991	0.984	0.947	0.961	0.961
2009	0.966	0.994	0.990	0.965	0.974	0.974
2010	0.978	0.996	0.993	0.977	0.982	0.983
Mean Efficiency	0.922	0.986	0.976	0.918	0.939	0.940

## Discussion

This study examined the efficiency of Malaysian banks listed in KLSE during 2005–2010 by applying a parametric frontier approach, SFA. The average technical efficiency of Malaysian banks listed in the KLSE was found to be 0.9463. About 94 percent of the banks were noted to have technical efficiency higher than the bank-industry average and about 6 percent of the banks in Malaysia listed in KLSE were observed to have less than the bank-industry average for technical efficiency. According to our results, RHBCAP seems to be the most efficient bank, while PBBANK appears to be the least efficient bank. Moreover, banks that made more deposits and less overhead expenses were found to be more efficient. We noted that the level of technical efficiency has increased over the reference period.

Efficiency estimation is useful for individual investment or loan decisions. Creditors and investors can critic the past performance and current position of banks by using efficiency results. Moreover, banks can improve their overall performance by taking decision based on efficiency results.

## References

[1] Olesen OB, Petersen NC, Lovell CAK (1996) Editors' introduction. Journal of Productivity Analysis 7 (2/3) 87–98.

[2] Aigner D, Lovell CAK, Schmidt P (1977) Formulation and Estimation of Stochastic Frontier Production Function Models. Journal of Econometrics 6: 21–37.

[3] Kirkley JE, Squires D, Strand IE (1995) Assessing Technical Efficiency in Commercial Fisheries: The Mid-Atlantic Sea Scallop Industry. American Journal of Agricultural Economics 77 3 686–697.

[4] Berger A, Humphrey D (1997) Efficiency of financial institutions: international survey and directions for future research. European Journal of Operation Research 98: 175–212.

[5] Sherman HD, Gold F (1985) Bank branch operating efficiency: Evaluation with data envelopment analysis. Journal of Banking and Finance 9: 279–315.

[6] Aly HY, Grabowski R, Pasurka C, Rangan N (1990) Technical, scale and allocative efficiencies in US banking: An empirical investigation. Review of Economics and Statistics 72: 211–218.

[7] Elyasiani E, Mehdian SM (1990) Efficiency in the commercial banking industry: A production frontier approach. Applied Economics 22: 539–551.

[8] Kwan SH, Eisenbeis RA (1996) An analysis of inefficiencies in banking: A stochastic cost frontier approach. Federal Reserve Bank of San Francisco Economic Review, No. 2.

[9] Worthington AC (1998) The determinants of non-bank financial institution efficiency: A stochastic cost frontier approach. Applied Financial Economics 8 3 279–289.

[10] Koetter M (2005) Measurement matters-input price proxies and bank efficiency in Germany, Discussion Paper Series 2: Banking and Financial Studies No. 01.

[11] Lozano-Vivas A (1997) Profit Efficiency of Spanish Savings Banks. European Journal of Operational Research 98 (2) 381–394.

[12] Berger AN, Humphrey DB (1992) Measurement and efficiency issues in commercial banking, in measurement issues in the service sector. Z. Griliches (ed.), NBER, Chicago.

[13] Boscia V (1999) The effect of deregulation on the Italian banking system: An empirical study. Research Papers in Banking and Finance, RP 98/14, School of Accounting, Banking and Economic, University of Wales, Bangor, Italy.

[14] Das A (1997) Technical, allocative and scale efficiency of public sector banks in India. RBI Occasional Papers 18 2&3 279–301.

[15] Shanmugam KR, Lakshmanasamy T (2001) Production frontier efficiency and measures: An analysis of the banking sector in India. Asian –African Journal of Economics and Econometrics 1 (2) 211–228.

[16] Kumar S, Satish V (2003) Technical efficiency, benchmarking and targets: A case study of Indian public sector banks. Prajnan 21 (4) 275–311.

[17] Mohan TTR, Ray S (2004) Comparing performance of public and private sector Banks: A revenue maximization approach. Economic and Political Weekly 39 (12) 1271–1275.

[18] Das A, Ashok N, Subhash R (2005) Liberalization, ownership and efficiency in Indian banking: A nonparametric analysis. Economic and Political Weekly 40 (12) 1190–1197.

[19] Kumbhakar SC, Subrata S (2003) Deregulation, ownership and productivity growth in the banking industry: Evidence from India. Journal of Money, Credit, and Banking 35 3 403–424.

[20] De PK (2004) Technical efficiency, ownership and reforms: An econometric study of Indian banking industry. Indian Economic Review 34 1 261–294.

[21] Sensarma R (2005) Cost and profit efficiency of Indian banks during 1986–2003: A stochastic frontier analysis. Economic and Political Weekly 40 12 Mar.19–25 1198–1209.

[22] Mahesh HP, Meenakshi R (2006) Liberalization and productive efficiency of Indian commercial banks: A stochastic frontier analysis. Manik Personal RePEc Archive Online at http://mpra.ub.uni-uenchen.de/827/MPRA Paper No. 827.

[23] Karim MZA (2001) Comparative bank efficiency across select ASEAN countries,. ASEAN Economic Bulletin 18 3 289–304.

[24] Fadzlan S, Muhd. Zulkhilbri AM (2005) Post-merger banks' efficiency and risks in emerging market: Evidence from Malaysia. The Icfai Journal of Bank Management 4 4 16–37.

[25] Lovell CAK (1993) Production frontiers and productive efficiency, in H.O. Fried, CAK Lovell, SS Schmidt (eds) The Measurement of Productive Efficiency. New York: Oxford University Press.

[26] Coelli TJ, Rao DSP, Battese GE (1998) An introduction to efficiency and productivity analysis. Kluwer Academic Publishers, Boston.

[27] Kumbhaker SC, Lovell CAK (2000) Stochastic Frontier Analysis. Cambridge: Cambridge University Press, UK.

[28] Battese GE, Coelli TJ (1992) Frontier production functions, technical efficiency and panel data: with application to paddy farmers in India. Journal of Productivity Analysis 3: 153–169.

[29] Coelli TJ (1996) A guide to FRONTIER version 4.1: A computer program for stochastic frontier production and cost function estimation. Mimeo, Department of Econometrics, University of New England, Armidale.

[30] Katib M, Nasser, Mathews K (2000) A Non-Parametric Approach to Efficiency Measurement in the Malaysian Banking Sector. The Singapore Economic Review 44: 89–114.

[31] Sufian F (2004) The Efficiency Effects of Bank Mergers and Acquisitions in Developing Economy: Evidence from Malaysia. International Journal of Applied Econometrics and Quantitative Studies 1 4 53–74.

